# Longitudinal co-development of social interaction, physical frailty, and subjective cognitive decline among community-dwelling older adults in Japan: a six-year parallel growth study

**DOI:** 10.3389/fpubh.2026.1913236

**Published:** 2026-09-09

**Authors:** Haotian Gao, Shuanghong Li, Ruifeng Zhao, Lujiao Huang, Jinrui Zhang, Yixin Sun, Meiling Qian, Mengxuan Wang, Maiko Shigeeda, Zhu Zhu, Dandan Jiao, Yang Liu, Mingyu Cui, Mengjiao Yang, Akihiro Kakuda, Yuko Sawada, Tokie Anme

**Affiliations:** 1 Doctoral Program in Medical Sciences, Graduate School of Comprehensive Human Sciences, University of Tsukuba, Tsukuba, Japan; 2 School of Nursing, Hangzhou Normal University, Hangzhou, China; 3 Department of Nursing, The First Affiliated Hospital, and College of Clinical Medicine, Henan University of Science and Technology, Luoyang, China; 4 Key Laboratory of Digital-Intelligent Disease Surveillance and Health Governance, North Sichuan Medical College, Nanchong, Sichuan, China; 5 Department of Nutrition and Food Hygiene, School of Public Health, Peking University, Beijing, China; 6 Department of Cardiovascular Surgery, Affiliated Hospital of North Sichuan Medical College, Nanchong, China; 7 Department of Physical Therapy, Morinomiya University of Medical Sciences, Osaka, Japan; 8 Faculty of Medicine, University of Tsukuba, Tsukuba, Japan

**Keywords:** community-dwelling older adults, healthy aging, Kihon checklist, latent growth curve model, physical frailty, social interaction, subjective cognitive decline

## Abstract

**Background:**

Population aging requires community-based approaches to identify multidomain risk patterns before severe disability or clinical cognitive impairment develops. Although social interaction, physical frailty, and subjective cognitive decline are relevant to healthy aging, their parallel longitudinal trajectories remain insufficiently characterized in community-dwelling older adults.

**Objective:**

This study examined the six-year co-development of social interaction, screening-defined physical frailty, and screening-defined subjective cognitive decline among community-dwelling older adults in Japan.

**Methods:**

This longitudinal cohort study analyzed 307 older adults (34.6% of 887 baseline-eligible participants) with valid measurements of all three focal variables in 2014, 2017, and 2020. Social interaction was measured using the Index of Social Interaction. Physical frailty (PF) was operationalized using the five-item motor domain of the Kihon Checklist (KCL), and screening-defined subjective cognitive decline (SCD) was operationalized using the three-item KCL Cognitive Function domain (KCL-CF). A Bayesian parallel-process latent-basis growth model was fitted, adjusting for baseline sociodemographic, lifestyle, and chronic disease factors.

**Results:**

The model showed acceptable posterior predictive fit. Social interaction scores decreased from 16.82 to 16.09, PF prevalence increased from 8.8 to 19.5%, and screening-defined SCD prevalence increased from 31.3 to 62.9%. A higher social-interaction intercept factor was associated with lower PF intercept liability (standardized estimate −0.282, 95% credible interval (CrI) −0.503 to −0.044) and lower SCD intercept liability (−0.433, −0.651 to −0.179). The social-interaction and PF slope factors were inversely associated (−0.490, −0.809 to −0.026), whereas slope associations involving SCD were imprecisely estimated.

**Conclusion:**

Social, physical, and subjective cognitive indicators showed distinct but interrelated longitudinal patterns. Repeated multidomain assessment may support community-based risk characterization and follow-up for healthy aging.

## Introduction

Frailty is a major expression of population aging and is commonly understood as a state of reduced physiological reserve and increased vulnerability to adverse outcomes. Early conceptualizations emphasized physical features such as weakness, exhaustion, slow walking speed, low activity, and weight loss, while deficit-accumulation approaches conceptualize frailty as a broader state arising from multiple age-related deficits (1–4). Clinical guidance also emphasizes the practical importance of identifying Physical frailty (PF) because it is dynamic and potentially modifiable (5). In parallel, the World Health Organization framework of healthy aging emphasizes functional ability as the result of intrinsic capacity interacting with the surrounding environment and care context, while intrinsic capacity frameworks highlight multiple domains such as locomotion, cognition, psychological capacity, vitality, and sensory function (6–8). These perspectives support examining frailty-related aging as a multidomain longitudinal process rather than as a single physical endpoint.

Physical frailty and cognitive vulnerability are closely interrelated in later life. Subjective cognitive decline (SCD) is not equivalent to dementia or objective cognitive impairment, but it captures self-perceived cognitive change and has been proposed as a clinically relevant early cognitive-risk construct (9, 10). Meta-analytic evidence suggests that subjective memory complaints and subjective cognitive decline are associated with increased risk of later mild cognitive impairment and dementia (11). Physical frailty is also linked to cognitive impairment and dementia risk through shared pathways such as reduced reserve, vascular burden, inflammation, mobility restriction, and psychological factors (12–14). However, less is known about whether physical frailty and subjective cognitive decline follow synchronized longitudinal trajectories in community-dwelling older adults.

Social interaction represents another domain of frailty-related aging. Social relationships and social participation are associated with survival, dementia risk, cognitive aging, and physical functioning in older adults (15–17). Social frailty research further suggests that reduced social resources and social participation can precede disability and later physical frailty (18, 19). In Japan, the Index of Social Interaction (ISI) was developed to assess multiple aspects of social interaction, including interaction with others, social curiosity, independence, participation, and feelings of safety, and has been used in longitudinal research on older adults (20, 21). Still, many studies examine social interaction as an exposure or covariate, rather than modeling it as one component of a broader social-physical-cognitive aging process.

The Kihon Checklist (KCL) provides a practical community screening tool for frailty-related risk in older adults in Japan. It has been validated for assessing frailty status and includes domains that capture physical function and cognitive concerns (22–24). It has also been used to predict long-term care risk in Japanese community settings (25). Because the Kihon Checklist is intended for community screening rather than clinical diagnosis, the present PF and SCD definitions should be interpreted as screening-based operationalizations rather than clinical diagnoses. Accordingly, SCD in the present study refers specifically to screening-defined SCD based on the KCL Cognitive Function domain (KCL-CF). This distinction is important when modeling longitudinal change in community-dwelling samples.

Latent growth curve models are suited to examining longitudinal change because they estimate both average trajectories and individual heterogeneity in change (26, 27). Parallel-process growth models extend this framework by estimating the interrelations among multiple longitudinal processes. For binary outcomes, categorical latent growth models can be specified using latent-response frameworks, and Bayesian estimation provides a flexible approach for estimating complex latent variable models with mixed continuous and categorical processes (28–30). Prior longitudinal studies have applied latent growth methods to frailty and cognition, social activity and cognition, and other psychosocial-cognitive aging processes (31–37). These studies support the utility of growth models for examining co-development among aging-related domains, but evidence remains limited regarding social interaction, physical frailty, and subjective cognitive decline as parallel processes.

The present study examined six-year parallel trajectories of social interaction, screening-defined physical frailty, and screening-defined subjective cognitive decline among community-dwelling older adults in Japan. Previous work from the same broader longitudinal community research program addressed a related but substantively different question. Cui et al. (37) analyzed 514 older adults across 2011, 2014, and 2017 and examined whether the three-item KCL subjective cognitive function score mediated the relationship between a five-item deficit-based social frailty score and five-item instrumental activities of daily living (IADL) functional status. In contrast, the present study analyzes 307 participants across 2014, 2017, and 2020 and treats the positively scored 18-item Index of Social Interaction, physical frailty (PF) operationalized from the five-item KCL motor domain, and screening-defined SCD based on the three-item KCL-CF as three parallel longitudinal processes. PF rather than IADL is the physical outcome, and the cognitive domain is not specified as a mediator. The present Bayesian mixed continuous/binary latent-basis model estimates initial levels, rates and relative timing of change, and cross-domain growth-factor associations rather than direct and indirect mediation effects. Thus, the practical question is also different: whether social, PF, and cognitive screening information provide redundant or complementary longitudinal information for repeated multidomain community follow-up. Earlier Community Empowerment and Care for Well-being and Healthy Longevity (CEC) work also addressed narrower pairwise questions; for example, Jiao et al. (21) examined changes in social relationships and subsequent physical-function decline across earlier waves using an exposure-outcome regression framework rather than a three-process parallel growth model. Cui et al. (37) and the present study arise from the same broader research program and share the 2014 and 2017 survey waves, so some participant-level overlap is possible; however, the published Cui et al. article does not report participant identifiers, and exact participant-level overlap cannot be determined from the publication alone. This analysis was designed to address a public health gap: although these domains are frequently examined as separate exposure-outcome relationships, less is known about whether they show synchronized or distinct longitudinal co-development in community settings. Clarifying these patterns may inform multidomain risk characterization and repeated community-based monitoring for healthy aging. Rather than testing a single exposure-outcome pathway, we aimed to characterize whether social interaction, PF, and screening-defined SCD followed coordinated or distinct trajectories and how their initial levels and rates of change were interrelated.

## Methods

### Study design and participants

**Figure 1:**
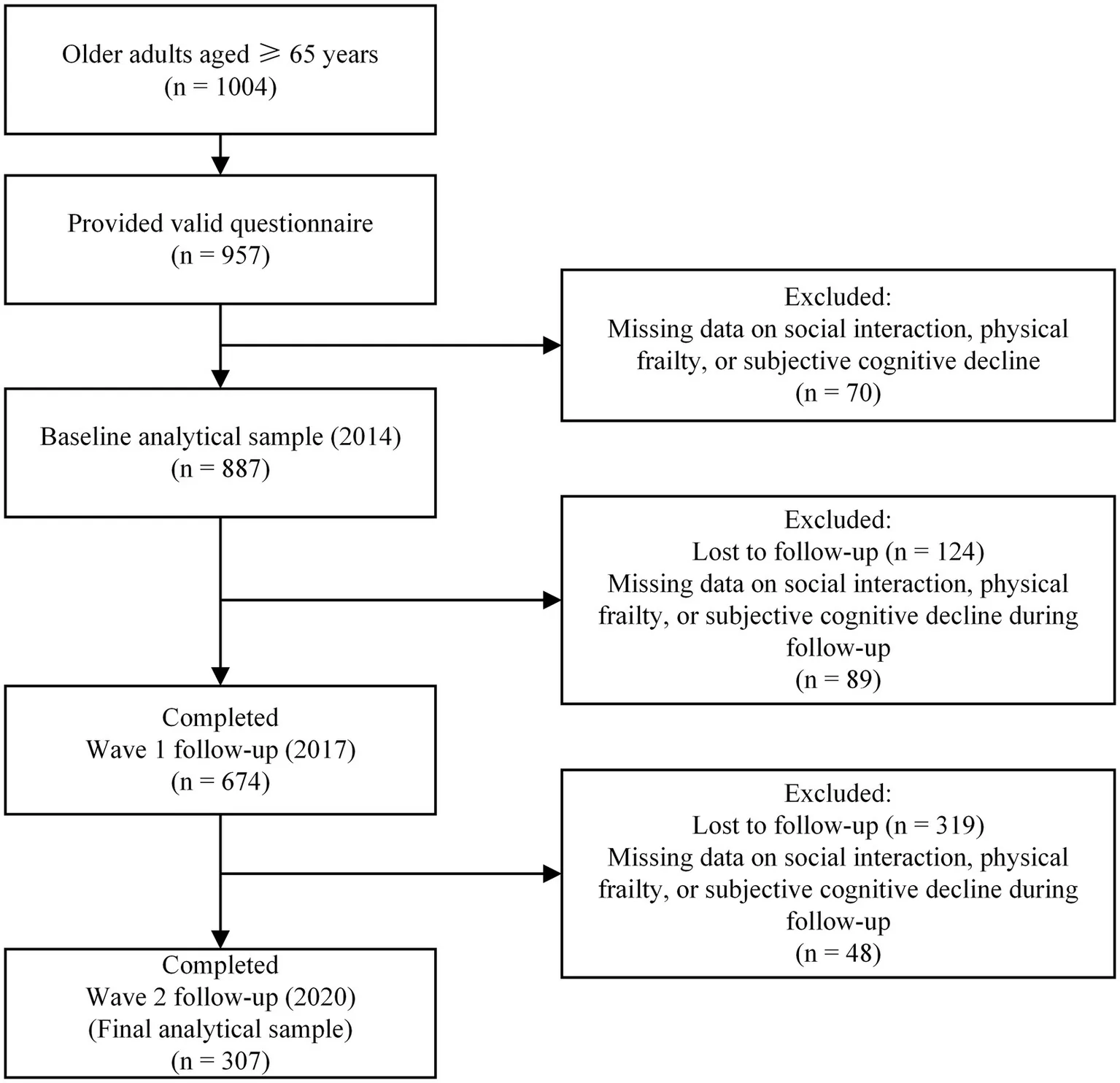
Participant flowchart. Numbers indicate participants remaining at each stage of sample selection. Exclusions during follow-up reflect loss to follow-up and missing data on the repeated measures used in the parallel-process latent growth curve model. The three focal measures were social interaction, screening-defined physical frailty, and screening-defined subjective cognitive decline (SCD) based on the three-item KCL Cognitive Function domain. KCL, Kihon Checklist; PF, Physical frailty; SCD, Screening-defined subjective cognitive decline.

This longitudinal cohort study used data from the Community Empowerment and Care for Well-being and Healthy Longevity (CEC) study, a community-based cohort study of older adults living in T village, central Japan. The CEC cohort is a community-wide municipal survey in which questionnaires are distributed to residents; assistance or interview support is available for residents who have difficulty completing the questionnaire, as described previously for this cohort (21). The primary assessment mode in the present waves was a self-administered questionnaire. Participants were observed in 2014, 2017, and 2020. Assessments used self-administered questionnaires, and the 2020 survey was conducted mainly in April 2020. The initial population consisted of 1,004 older adults aged 65 years or older. Among them, 957 returned valid questionnaires. After excluding participants with missing baseline data on social interaction, physical frailty, or subjective cognitive decline, 887 participants formed the baseline analytical sample. From 2014 to 2017, 213 participants were not retained because of loss to follow-up (*n* = 124) or missing required follow-up data (*n* = 89), leaving 674 participants. From 2017 to 2020, a further 367 were not retained because of loss to follow-up (*n* = 319) or missing required follow-up data (*n* = 48). The final three-wave analytical sample therefore included 307 participants. The participant flow is shown in Figure 1.

The available analytic dataset did not provide verified mutually exclusive subreasons for loss to follow-up such as death, relocation, or refusal; therefore, only the observed categories of loss to follow-up and missing required follow-up data are reported.

This study was approved by the Ethics Committee of the University of Tsukuba, Japan (approval No. 1331–7). The study used anonymized secondary data provided under an approved collaborative research framework. Consent procedures were approved by the Ethics Committee of the University of Tsukuba, Japan.

### Measures

Social interaction was assessed using the 18-item Index of Social Interaction. The total score was retained on its original 0–18 scale, with higher scores indicating greater social interaction (20). The original scale was retained in the latent growth curve model because wave-specific standardization would remove meaningful longitudinal mean differences.

Physical frailty (PF) was operationalized using the five-item motor domain of the Kihon Checklist (KCL). The five motor-domain items assessed stair climbing without a handrail or wall, standing from a chair without aids, walking continuously for 15 min, falls in the previous year, and fear of falling while walking. For the first three items, “No” was coded as unfavorable; for the two fall-related items, “Yes” was coded as unfavorable, giving a motor-domain score from 0 to 5. Participants with three or more unfavorable responses in the five-item KCL motor domain were classified as having PF, consistent with the established KCL motor-domain screening criterion (23, 38). PF is the study outcome, whereas the KCL motor domain is the measurement domain used to operationalize it; this screening-based definition should not be interpreted as a clinical frailty phenotype or diagnosis.

Screening-defined subjective cognitive decline (SCD) was operationalized using the three-item Cognitive Function domain of the Kihon Checklist (KCL-CF). The items assessed whether family or friends pointed out memory loss, whether the participant could make a telephone call by looking up telephone numbers, and whether the participant sometimes did not know the current date. Unfavorable responses were “Yes,” “No,” and “Yes,” respectively, yielding a KCL-CF score from 0 to 3. One or more unfavorable responses (KCL-CF ≥ 1) were classified as screening-defined SCD. Tomata et al. (39) reported predictive validity of the same ≥1 cutoff for incident dementia. Previous studies of community-dwelling Japanese older adults have also used the KCL-CF to operationalize SCD, including the same ≥1 cutoff (40, 41). In the present study, this definition is used as a self-reported community-screening operationalization and should not be interpreted as equivalent to formal research criteria for SCD or as a clinical diagnosis of SCD, mild cognitive impairment (MCI), dementia, or objective cognitive impairment.

Baseline covariates included age, sex, employment status, subjective economic status, smoking status, alcohol consumption, and chronic disease count. Age and chronic disease count were treated as continuous covariates, whereas the other covariates were treated as categorical or binary baseline covariates. Age and chronic disease count were standardized in the model to improve numerical stability and interpretability of covariate effects.

### Statistical analysis

We first summarized observed trajectories of social interaction, physical frailty, and subjective cognitive decline across 2014, 2017, and 2020. Social interaction was summarized as mean and standard deviation, whereas physical frailty and subjective cognitive decline were summarized as the number and percentage of participants classified as having the screening-defined risk state.

The primary analysis used a Bayesian parallel-process latent-basis growth model. Social interaction was modeled as a continuous repeated outcome.

$$SI_{\mathrm{it}}=\eta_{0i}^{\mathrm{SI}}+\lambda_{t}^{\mathrm{SI}}\eta_{1i}^{\mathrm{SI}}+\varepsilon_{\mathrm{it}}^{\mathrm{SI}}$$

$$Y_{\mathrm{it}}^{*k}=\eta_{0i}^{k}+\lambda_{t}^{k}\eta_{1i}^{k}+\varepsilon_{\mathrm{it}}^{k}$$

$$Y_{\mathrm{it}}^{k}=\{\begin{array}{ll} 1, & Y_{\mathrm{it}}^{*k}>0,\\ 0, & Y_{\mathrm{it}}^{*k}\leq 0. \end{array}$$

$$\eta_{0i}^{k}=\alpha_{0}^{k}+\gamma_{0}^{k\prime }X_{i}+\zeta_{0i}^{k}$$

$$\eta_{1i}^{k}=\alpha_{1}^{k}+\gamma_{1}^{k\prime }X_{i}+\zeta_{1i}^{k}$$

For the binary processes, Y* denotes the continuous latent response underlying the observed binary status; PF and screening-defined SCD were coded 0 = absent and 1 = present. PF and SCD were modeled as binary outcomes using a probit latent-response framework; their growth factors therefore represent change in the underlying latent liability for the corresponding screening-defined status. Higher latent liability corresponded to a higher probability of being classified as having PF or screening-defined SCD (28, 30). The binary indicators were coded as 0 = absence and 1 = presence of the screening-defined risk state; therefore, higher latent liability corresponded to a higher probability of being classified as having physical frailty or subjective cognitive decline. Thresholds for the binary repeated indicators were fixed to zero so that longitudinal change was represented by the growth factors rather than by freely varying thresholds. Fixing the thresholds at zero was an identification constraint on the probit latent-response scale and does not imply 50% observed prevalence. A model with freely varying thresholds would redistribute longitudinal change between thresholds and growth factors and would therefore have a different parameterization and interpretation. Accordingly, growth-factor means for physical frailty and subjective cognitive decline were interpreted on the latent-liability scale and were considered together with the observed prevalence trajectories and latent-basis loadings. For each process, the 2014 loading was fixed at 0 and the 2020 loading was fixed at 1. The 2017 loading was freely estimated, allowing the model to characterize whether the change occurred mainly during 2014–2017 or 2017–2020.

All six growth factors, consisting of intercept and slope factors for social interaction, physical frailty, and subjective cognitive decline, were estimated jointly. All 15 pairwise covariances among the six growth factors were estimated. Residual variances for the continuous ISI indicators were estimated freely; no additional same-wave residual covariances between domains were specified; and the residual scale for the binary outcomes followed the Mplus probit parameterization. The model estimated level-level associations, slope-slope associations, and level-change associations among these growth factors. Baseline covariates were specified as predictors of all growth factors. The final Mplus prior specification was as follows: the freely estimated 2017 loadings for PF and SCD used N(0, 5) defaults; the freely estimated ISI loading and unrestricted location/regression parameters used diffuse N(0, infinity) defaults; ISI residual variances used IG(−1, 0); the six-growth-factor residual covariance matrix used IW(I6, 7); and the seven baseline-covariate covariance matrix used IW(0, −8). The only non-default prior was N(0.5, 1.0) for the PF slope-factor mean. The grouped prior specification from the final Mplus output is reproduced in Supplementary Text S1. The model was estimated in Mplus version 8.3 using four Markov chain Monte Carlo (MCMC) chains, 320,000 iterations, thinning of 10, and random seed 2026 (29, 30). The model terminated normally. TECH8 maximum potential scale reduction factor (PSR) values were 1.021 at 10,000 iterations, 1.005 at 20,000, 1.002 at 64,000, 1.009 at 160,000, and 1.002 at 320,000. Posterior predictive checking gave a posterior predictive p-value (PPP) of 0.587 and a 95% observed-minus-replicated chi-square interval of −53.58 to 42.80. Because fixed iterations were requested, Mplus also printed a recommendation to increase FBITERATIONS by at least a factor of two as an additional convergence check; that additional run was not performed. Point estimates are reported as posterior medians with 95% credible intervals.

To evaluate selective retention, baseline characteristics were compared between participants retained in the common three-wave analytical sample (n = 307) and baseline-eligible participants who were not retained through all three waves (n = 580). Continuous variables were summarized using means and standard deviations and categorical variables using counts and percentages based on available observations. Standardized mean differences (SMDs) were calculated to describe the magnitude and direction of between-group differences. Missing baseline employment, economic-status, smoking, or alcohol data did not trigger additional listwise deletion in the Bayesian model because the means, variances, and covariance structure of the baseline covariates were estimated jointly.

## Results

### Participant characteristics and observed trajectories

The final analytical sample included 307 community-dwelling older adults. Mean baseline age was 71.64 years (SD 5.61), and 168 participants (54.7%) were women. At baseline, 142 participants (46.3%) were working and the mean chronic disease count was 1.36 (SD 1.04). The prevalence of screening-defined physical frailty was 8.8%, and the prevalence of screening-defined subjective cognitive decline was 31.3% at baseline (Table 1).

**Table 1:** Characteristics of the three-wave analytical sample and baseline retention comparison.

Variable	Mean ± SD
Panel A: Continuous	
Baseline age	71.64 ± 5.61
Chronic disease count	1.36 ± 1.04
Social interaction T1 (2014)	16.82 ± 1.75
Social interaction T2 (2017)	16.52 ± 2.03
Social interaction T3 (2020)	16.09 ± 2.44

Variable	Category	*n* (%)
Panel B: Categorical covariates and binary outcomes		
Sex	Male	139 (45.3)
	Female	168 (54.7)
Employment status	Working	142 (46.3)
	Not working	131 (42.7)
	Missing	34 (11.1)
Subjective economic status	Good	246 (80.1)
	Not good	49 (16.0)
	Missing	12 (3.9)
Smoking status	Current smoker	27 (8.8)
	Non-smoker	265 (86.3)
	Missing	15 (4.9)
Alcohol consumption	Current drinker	109 (35.5)
	Non-drinker	191 (62.2)
	Missing	7 (2.3)
Physical frailty at T1 (2014)	Present	27 (8.8)
	Not present	280 (91.2)
Physical frailty at T2 (2017)	Present	38 (12.4)
	Not present	269 (87.6)
Physical frailty at T3 (2020)	Present	60 (19.5)
	Not present	247 (80.5)
Subjective cognitive decline at T1 (2014)	Present	96 (31.3)
	Not present	211 (68.7)
Subjective cognitive decline at T2 (2017)	Present	184 (59.9)
	Not present	123 (40.1)
Subjective cognitive decline at T3 (2020)	Present	193 (62.9)
	Not present	114 (37.1)

Characteristic	Not retained	Retained	SMD
Panel C: Baseline comparison of participants retained and not retained in the three-wave analytical sample			
Baseline age, years	75.68 ± 7.66	71.64 ± 5.61	−0.601
Chronic disease count	1.48 ± 1.23	1.36 ± 1.04	−0.103
ISI score	15.72 ± 2.76	16.82 ± 1.75	0.473
Female	303/580 (52.2%)	168/307 (54.7%)	0.050
Working	178/491 (36.3%)	142/273 (52.0%)	0.317
Good subjective economic status	443/529 (83.7%)	246/295 (83.4%)	−0.010
Current smoking	57/501 (11.4%)	27/292 (9.2%)	−0.070
Current drinking	153/532 (28.8%)	109/300 (36.3%)	0.162
PF	136/577 (23.6%)	27/307 (8.8%)	−0.401
Screening-defined SCD	244/580 (42.1%)	96/307 (31.3%)	−0.224

Data are presented as mean ± standard deviation (SD) for continuous variables and *n* (%) for categorical variables. Social interaction was assessed using the 18-item Index of Social Interaction, which ranges from 0 to 18, with higher scores indicating greater social interaction. Physical frailty was operationalized as three or more unfavorable responses in the five-item motor domain of the Kihon Checklist. Screening-defined SCD was operationalized as one or more unfavorable responses in the three-item KCL Cognitive Function domain (KCL-CF). Percentages for baseline categorical covariates were calculated using the full analytic sample (*n* = 307), with missing values shown explicitly where applicable. Abbreviations: SD, standard deviation; T1, baseline in 2014; T2, 2017 follow-up; T3, 2020 follow-up; ISI, Index of Social Interaction; KCL, Kihon Checklist; KCL-CF, KCL Cognitive Function domain; PF, physical frailty; SCD, screening-defined subjective cognitive decline; SMD, standardized mean difference. Categorical denominators are based on available observations; Supplementary Table S1 retains the full missing-data detail. SMD direction is retained minus not retained.

Compared with participants who were not retained, retained participants were younger (71.64 vs. 75.68 years; SMD = −0.601), had higher baseline social interaction (16.82 vs. 15.72; SMD = 0.473), were more likely to be working (52.0% vs. 36.3% among participants with available employment data; SMD = 0.317), and had lower PF prevalence (8.8% vs. 23.6%; SMD = −0.401) and screening-defined SCD (31.3% vs. 42.1%; SMD = −0.224). Full comparisons and available-data denominators are shown in Table 1 and Supplementary Table S1.

**Figure 2:**
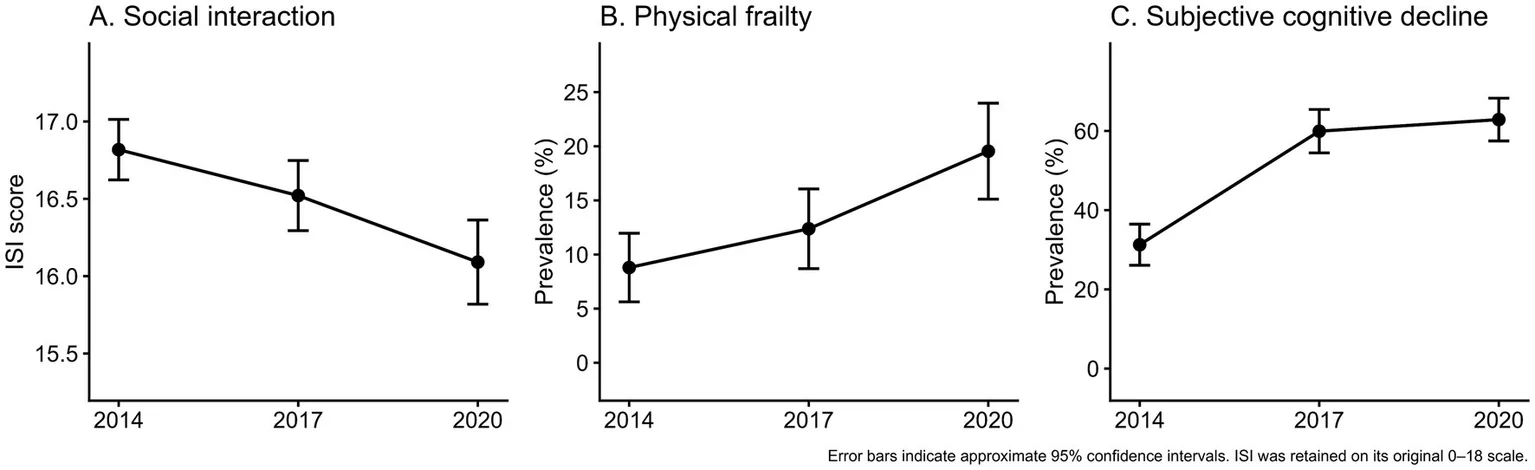
Observed trajectories of social interaction, physical frailty, and subjective cognitive decline. Note: Points represent observed means for social interaction and observed prevalence for physical frailty and screening-defined SCD. Error bars indicate approximate 95% confidence intervals. The Index of Social Interaction (ISI) ranges from 0 to 18 and was retained on its original scale. Physical frailty was defined as three or more unfavorable responses in the five-item Kihon Checklist (KCL) motor domain. Screening-defined SCD was defined as one or more unfavorable responses in the three-item KCL Cognitive Function domain. Physical frailty and subjective cognitive decline are shown as percentages classified as screening-defined risk states. Error bars represent 95% confidence intervals. For continuous ISI means, intervals were calculated as *mean* ± 1.96 × *standard error*; for binary prevalences, normal-approximation binomial intervals were calculated as *p* ± 1.96√[*p*(1-*p*)/*n*].

Observed social interaction scores showed a mild decline over time, from 16.82 (SD 1.75) in 2014 to 16.52 (SD 2.03) in 2017 and 16.09 (SD 2.44) in 2020. Physical frailty prevalence increased from 8.8% in 2014 to 12.4% in 2017 and 19.5% in 2020. Subjective cognitive decline prevalence increased from 31.3% in 2014 to 59.9% in 2017 and 62.9% in 2020 (Figure 2). ISI scores showed substantial upper-end concentration, with 43.7% of participants scoring the maximum value of 18 in 2014, 40.7% in 2017, and 30.3% in 2020.

### Model fit and trajectory parameters

The Bayesian parallel-process latent-basis growth model showed acceptable posterior predictive fit and satisfactory convergence. The posterior predictive *p*-value was 0.587, and the 95% credible interval for the observed-replicated chi-square difference ranged from −53.58 to 42.80, including zero. The final potential scale reduction factor was 1.002 (Table 2).

**Table 2:** Bayesian model fit, trajectory parameters, and standardized cross-domain associations in the parallel-process LGCM.

Index	Value
Panel A: Model fit and convergence diagnostics	
Number of observations	307
Estimator	Bayesian estimation
Link function	Probit
Number of chains	4
Iterations	320,000
Thinning	10
Number of free parameters	110
Posterior predictive *p*-value (PPP)	0.587
95% CrI for observed–replicated *χ*^2^ difference	−53.58 to 42.80
Final PSR	1.002

Parameter	Estimate	95% CrI
Panel B: Latent-basis loadings and posterior medians of growth factors		
SI loading at T2 (T1 = 0, T3 = 1)	0.521	0.377 to 0.673
PF loading at T2 (T1 = 0, T3 = 1)	0.299	0.090 to 0.557
SCD loading at T2 (T1 = 0, T3 = 1)	0.834	0.638 to 1.057
i-SI	16.504	15.571 to 17.438
s-SI	−0.932	−1.981 to 0.117
i-PF	−2.599	−4.542 to −1.025
s-PF	0.023	−1.594 to 1.613
i-SCD	−0.772	−1.782 to 0.200
s-SCD	1.535	0.276 to 2.848

Growth-factor association	Standardized β	95% CrI
Panel C. Key standardized cross-domain associations among growth factors		
i-SI with i-PF	−0.282	−0.503 to −0.044
i-SI with i-SCD	−0.433	−0.651 to −0.179
i-PF with i-SCD	0.130	−0.262 to 0.509
i-SI with s-PF	−0.741	−0.910 to −0.464
i-SI with s-SCD	−0.081	−0.498 to 0.414
i-SCD with s-PF	0.439	0.020 to 0.755
s-SI with s-PF	−0.490	−0.809 to −0.026
s-SI with s-SCD	0.069	−0.441 to 0.543
s-PF with s-SCD	0.210	−0.424 to 0.686

Panel A presents Bayesian model fit and convergence diagnostics for the covariate-adjusted parallel-process latent growth curve model. Panel B presents latent-basis loadings and posterior medians of growth factors. The T2 loading describes the relative position of the 2017 assessment within the total model-implied change from 2014 to 2020, with T1 fixed at 0 and T3 fixed at 1; its 95% credible interval should be considered when interpreting the timing of change. Physical frailty and subjective cognitive decline were modeled as binary screening-defined risk states using a probit latent-response framework. The binary indicators were coded as 0 = absence and 1 = presence of the screening-defined risk state; therefore, higher latent liability corresponded to a higher probability of being classified as having the corresponding risk state. Thresholds for the binary repeated indicators were fixed to zero, and longitudinal change was represented by the growth factors. Growth-factor estimates for physical frailty and subjective cognitive decline are on the latent-liability scale and should be interpreted together with the observed prevalence trajectories. Panel C presents standardized cross-domain associations among growth factors in the same order as the main forest plot. Estimates are reported with 95% credible intervals (CrIs). LGCM, latent growth curve model; SI, social interaction; PF, physical frailty; KCL, Kihon Checklist; SCD, screening-defined subjective cognitive decline based on the three-item KCL Cognitive Function domain; i, intercept growth factor; s, slope growth factor; PPP, posterior predictive p-value; PSR, potential scale reduction factor; CrI, credible interval.

Latent-basis loadings indicated different timing of change across domains. The estimated 2017 loading was 0.521 for social interaction, 0.299 for physical frailty, and 0.834 for subjective cognitive decline. These estimates suggested different timing of change across the three processes. Social interaction showed a gradual decline. For subjective cognitive decline, most of the estimated increase in latent liability had occurred by 2017. The 2017 loading was 0.521 (95% credible interval (CrI) 0.377 to 0.673) for social interaction, 0.299 (0.090 to 0.557) for PF, and 0.834 (0.638 to 1.057) for SCD. Because the SCD interval extended above 1.0, the latent-basis estimates do not establish that SCD changed earlier than PF; the model-implied timing estimates should be interpreted separately from the observed prevalence trajectories. The posterior slope medians were −0.932 (95% CrI −1.981 to 0.117) for social interaction, 0.023 (−1.594 to 1.613) for PF, and 1.535 (0.276 to 2.848) for SCD; these are model-scale growth parameters rather than direct observed changes in scores or prevalence.

### Associations among growth factors

Higher stable social interaction was associated with lower stable liability for physical frailty [standardized estimate −0.282, 95% credible interval (CrI) −0.503 to −0.044] and subjective cognitive decline (standardized estimate −0.433, 95% CrI −0.651 to −0.179). The level-level association between physical frailty and subjective cognitive decline was not clearly supported (standardized estimate 0.130, 95% CrI −0.262 to 0.509).

Level-change associations provided additional information. Higher stable social interaction was associated with a more favorable physical frailty slope factor (standardized estimate −0.741, 95% CrI −0.910 to −0.464), but not clearly with the subjective cognitive decline slope factor (standardized estimate −0.081, 95% CrI −0.498 to 0.414). Higher stable subjective cognitive decline liability was associated with a less favorable physical frailty slope factor (standardized estimate 0.439, 95% CrI 0.020 to 0.755).

**Figure 3:**
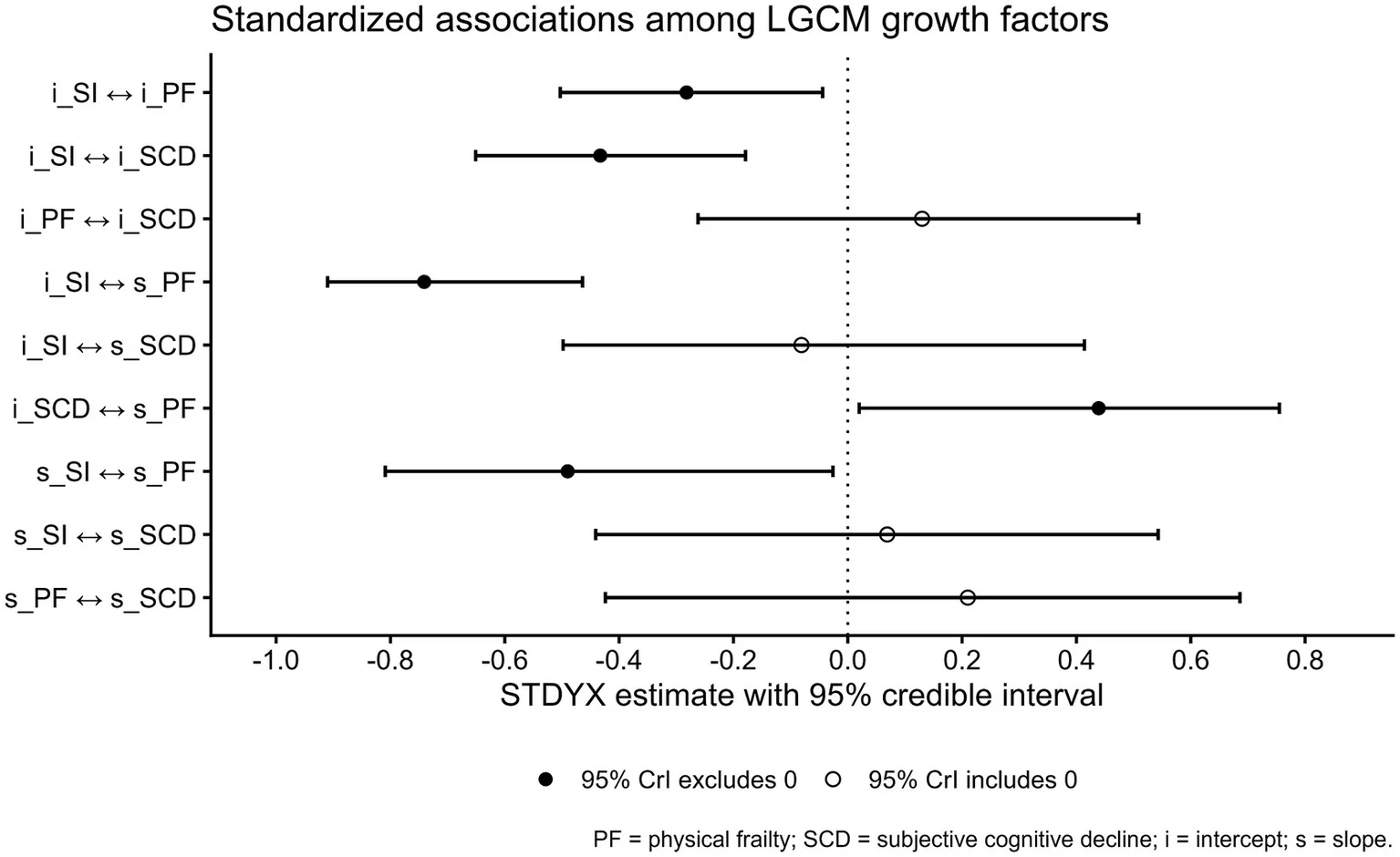
Standardized cross-domain growth-factor associations in the Bayesian parallel-process latent growth curve model. Note: Dots represent standardized posterior medians and horizontal lines represent 95% credible intervals. Filled circles indicate intervals excluding zero; open circles indicate intervals including zero. The dotted vertical line indicates zero. These estimates correspond to the standardized cross-domain associations shown in Table 2. SI, social interaction; PF, physical frailty; SCD, screening-defined subjective cognitive decline; i, intercept growth factor; s, slope growth factor.

Among slope-slope associations, only the association between social interaction and physical frailty was clearly supported. The standardized association between the social interaction slope and physical frailty slope was −0.490 (95% CrI −0.809 to −0.026), whereas the associations between the social interaction slope and subjective cognitive decline slope (standardized estimate 0.069, 95% CrI −0.441 to 0.543) and between the physical frailty slope and subjective cognitive decline slope (standardized estimate 0.210, 95% CrI −0.424 to 0.686) were not clearly supported. These associations are summarized in Table 2 and Figure 3.

Among the baseline covariates, older age was associated with lower social-interaction intercept and slope factors and higher PF intercept and slope factors. Working status was associated with higher social-interaction intercept and slope factors, and chronic disease count was associated with a higher PF intercept factor. Full covariate estimates and growth-factor residual variances are shown in Supplementary Table S2.

## Discussion

This study examined the six-year co-development of social interaction, screening-defined physical frailty, and screening-defined subjective cognitive decline among community-dwelling older adults in Japan. From an aging and public health perspective, three findings are central. First, the domains showed different observed and model-implied temporal patterns, but the latent-basis timing estimates were uncertain, particularly for screening-defined SCD. Most of the estimated increase in subjective cognitive decline liability had occurred by 2017, whereas the observed prevalence of physical frailty increased more prominently during the later interval, and social interaction showed a mild decline. Second, stable social interaction was inversely associated with stable physical frailty and subjective cognitive decline liability. Third, the interrelations among changes were domain-specific: the social interaction and PF slope factors were inversely associated (−0.490, 95% CrI −0.809 to −0.026), whereas slope associations involving screening-defined SCD were imprecisely estimated. These findings suggest that social, physical, and subjective cognitive indicators may provide complementary information for community-based risk characterization rather than representing a single synchronized aging process.

The finding that physical frailty and subjective cognitive decline followed different temporal patterns is consistent with the view that frailty-related aging is multidomain and heterogeneous. Frailty research has long emphasized increased vulnerability to adverse outcomes, but contemporary models also recognize multiple physical, cognitive, psychological, and social domains (1–8). Our results suggest that even within a single community cohort, social interaction, physical frailty, and subjective cognitive decline do not necessarily deteriorate as one synchronized process.

Possible differences in timing between PF and screening-defined SCD should be interpreted cautiously because the cognitive-domain 2017 loading was imprecisely estimated and the two screening measures also have different measurement properties. Subjective cognitive decline was based on self-reported cognitive concerns and cognitively oriented daily function items, whereas physical frailty was based on more overt locomotor and physical function difficulties. Accordingly, differences between the PF and cognitive trajectories may reflect both true differences in longitudinal development and differences in measurement sensitivity; the present model does not establish a temporal sequence between them.

Our results extend previous longitudinal work linking physical frailty and cognitive function. Peng and colleagues used latent growth curve models to show that physical frailty was related to cognitive function level and change among older Chinese adults (31). Other longitudinal growth modeling studies have examined social activity, physical function, cognitive function, and psychological symptoms as jointly developing processes in older adults (32–36). The present study differs by focusing on screening-defined physical frailty and subjective cognitive decline and by including social interaction as a parallel social process. Although our sample was smaller and our cognitive indicator was subjective rather than objective, the present findings are consistent with the broader view that physical and cognitive vulnerability should be examined as interrelated processes rather than as isolated outcomes.

Stable social interaction was inversely associated with stable physical frailty and subjective cognitive decline liability. This finding is consistent with evidence linking social relationships, social participation, and social frailty with survival, dementia risk, physical function, and disability (15–21). It is also consistent with a recent Japanese latent growth curve study showing links among social frailty, subjective cognitive function, and functional status trajectories (37). However, our findings should not be interpreted as evidence that social interaction directly prevents physical frailty or subjective cognitive decline. Social interaction should therefore be viewed as a contextual indicator within multidomain assessment rather than as a demonstrated intervention target. The model estimated associations among growth factors in an observational cohort, and residual confounding and selection processes remain possible. A more cautious interpretation is that social interaction represents a stable social-contextual dimension that co-occurs with lower frailty-related physical and subjective cognitive risk.

The slope-slope findings were also informative. Rather than showing a fully synchronized three-domain pattern, the evidence for coupled change was specific to social interaction and physical frailty. A more favorable social interaction trajectory was associated with a more favorable physical frailty slope factor, whereas slope-slope associations involving subjective cognitive decline were not clearly supported. This pattern suggests that social and physical frailty-related changes may be more closely coupled than subjective cognitive change over the same six-year period. The level-change associations further suggested that higher stable social interaction was linked to a more favorable physical frailty slope factor, whereas higher stable subjective cognitive decline liability was linked to a less favorable physical frailty slope factor. These findings may reflect broader differences in reserve, engagement, health behavior, or functional vulnerability, but they do not establish causal ordering.

The public-health value of these findings lies in repeated multidomain risk characterization rather than evidence of an intervention effect. First, repeated multidomain assessment may be more informative than one-time screening because the timing and measurement sensitivity of the social, physical, and subjective cognitive indicators differed across waves. Second, when a new or persistent positive screen or concern appears in one domain, the other domains should be reassessed rather than inferred from that single result; repeated multidomain follow-up can determine whether risk remains isolated or becomes co-occurring across social, PF, and cognitive domains. Third, social interaction may be considered alongside physical and cognitive screening indicators, not as a substitute for them, but as part of a broader community-based healthy aging monitoring framework. From a public health perspective, the value of this approach lies in identifying heterogeneous patterns of co-development among social, physical, and subjective cognitive risk indicators rather than establishing a single directional pathway. These implications align with the broader shift toward functional and multidomain assessment in healthy aging and intrinsic capacity frameworks (6–8).

The 2020 survey was conducted mainly in April 2020, during the early phase of the COVID-19 pandemic. No COVID-19 cases had been reported in T village by the end of April 2020, suggesting that the direct local impact of infection was limited during the main survey period. However, by that time, national and prefectural emergency measures restricting non-essential outings and some community activities were already in place and may have influenced social interaction, daily activities, and self-reported cognitive concerns (42). Because pandemic-specific exposure variables were not collected and all participants were assessed under the same calendar-time conditions in 2020, the observed changes between 2017 and 2020 cannot be statistically separated into aging-related and pandemic-related components. We therefore do not attribute the 2017–2020 changes specifically to COVID-19.

Several limitations should be acknowledged. First, the study used a single community cohort in Japan, and the analytical sample was modest, which may limit generalizability and statistical precision. The model included 110 free parameters in a three-wave analytical sample of 307 participants, and several posterior intervals—particularly slope associations involving screening-defined SCD—were wide; intervals containing zero are therefore interpreted as imprecise estimates rather than evidence that the longitudinal processes are independent. Second, attrition across the six-year follow-up was substantial.

Retention was selective. Compared with participants who were not retained, those in the three-wave analytical sample were younger, had higher baseline social interaction, were more likely to be working, and had lower PF and screening-defined SCD. The retained-versus-non-retained comparison describes the direction and magnitude of selection but does not statistically correct possible attrition bias; the longitudinal estimates should therefore be interpreted as characterizing participants who remained observable across all three waves rather than the full baseline cohort.

No all-available-data sensitivity model was added. The 580 participants not retained in the common three-wave sample include both complete loss to follow-up and wave-specific missingness across one continuous and two binary focal processes. The reported Bayesian probit latent-basis model therefore cannot be extended to N = 887 as a like-for-like sensitivity analysis simply by changing the sample size. full-information maximum likelihood (FIML) would require a different estimation framework, while a Bayesian all-available analysis, although possible in principle, would require a different missing-data specification and would target baseline-cohort trajectories under assumptions about unobserved follow-up outcomes. For participants lost after baseline or an intermediate wave, slope and cross-domain change estimates would consequently become substantially model-dependent. Because these assumptions are not testable and retention was demonstrably selective, the manuscript does not present an N = 887 model as an equivalent robustness check or as a correction for attrition bias. Instead, selective retention is quantified directly, and interpretation is restricted to participants observed across all three waves. Accordingly, the findings should be interpreted as characterizing temporal co-development among older adults with valid repeated measurement across all three focal domains, rather than as population-level transition estimates for the entire baseline cohort. Third, a possible ceiling effect in social interaction should be considered. ISI scores were concentrated at the maximum value of 18 in 43.7% of participants in 2014, 40.7% in 2017, and 30.3% in 2020. This may indicate that the baseline distribution of social interaction was restricted among relatively well-functioning community-dwelling older adults. Restricted variability at the upper end of the scale could have limited the detection of longitudinal change and attenuated slope-related associations with physical frailty and subjective cognitive decline. Accordingly, the weaker associations involving changes in social interaction should be interpreted with caution. Fourth, although the model adjusted for baseline sociodemographic, lifestyle, and chronic disease factors, residual confounding remains possible. Unmeasured or time-varying factors such as depressive symptoms, sensory impairment, health service use, social support quality, objective cognitive performance, and incident disease may have influenced both social interaction and frailty-related indicators. Fifth, PF was dichotomized from the 0–5 KCL motor-domain score and screening-defined SCD from the 0–3 KCL-CF score using established screening cutoffs (23, 38–41). This preserves the community-screening interpretation but does not retain the full gradation of the original scores and may reduce statistical information. Neither binary classification should be interpreted as a clinical diagnosis. In particular, screening-defined SCD in this study reflects a brief self-reported KCL-based measure and may also be influenced by psychological distress, health perception, or awareness of functional change. Sixth, subjective cognitive decline was self-reported and may reflect psychological distress, health perception, or awareness of functional change in addition to cognitive risk. The available measures and assessment intervals reflected the design of the CEC cohort. Although the selected indicators were theoretically aligned with multidomain healthy aging and community screening, the study could not incorporate all potentially relevant domains, such as objective cognitive performance, depressive symptoms, sensory function, health service use, or detailed social support quality. Finally, with three waves, the latent-basis model could characterize broad timing differences but could not evaluate more complex nonlinear patterns, and the observational design precludes causal inference.

## Conclusion

In this six-year cohort of community-dwelling older adults in Japan, social interaction, screening-defined physical frailty, and screening-defined subjective cognitive decline showed distinct but interrelated longitudinal patterns. The latent-basis timing estimates were uncertain, particularly for screening-defined SCD, and do not establish a temporal ordering between the cognitive and PF processes. Higher stable social interaction was associated with lower stable liability for physical frailty and subjective cognitive decline, and social interaction change was inversely associated with physical frailty change. These findings should be interpreted as evidence of temporal co-development among social, physical, and subjective cognitive screening indicators, not as evidence of causal ordering. Repeated multidomain assessment may support community-based risk characterization and follow-up strategies for healthy aging, particularly when social, physical, and subjective cognitive concerns emerge in different temporal patterns.

## Data Availability

The datasets analyzed for this study are not publicly available because they contain confidential community survey data from the Community Empowerment and Care for Well-being and Healthy Longevity (CEC) study and are subject to research governance and ethics restrictions. Data access is subject to approval by the relevant data custodian and ethics committee. Requests to access these datasets should be directed to TA, tokieanme@gmail.com.
