# Supplementary material for: Longitudinal co-development of social interaction, physical frailty, and subjective cognitive decline among community-dwelling older adults in Japan: a six-year parallel growth study
Source: Front Public Health. 2026 Sep 9;14:1913236. doi: 10.3389/fpubh.2026.1913236 (PMC13603030; doi:10.3389/fpubh.2026.1913236)
Supplement: Supplementary file 1 [file Supplementary_file_1.DOCX]

**Supplementary Text S1. Mplus Syntax for the Bayesian Parallel-Process Latent-Basis Growth Model**

This syntax corresponds to the final reported model. The file path is shown generically for portability. The internal variable labels scd14/scd17/scd20 correspond to screening-defined SCD based on the three-item KCL Cognitive Function domain in the revised manuscript. They do not denote a formal clinical diagnosis of SCD.

Mplus version 8.3; estimator = BAYES; four chains; 320,000 iterations; thinning = 10; seed = 2026. Mplus default priors were used except for the PF slope-factor mean, for which N(0.5, 1.0) was specified.

TITLE:

Bayesian parallel-process latent-basis growth model: raw ISI, binary PF and screening-defined SCD.

DATA:

FILE IS "307bina.dat";

FORMAT IS FREE;

VARIABLE:

NAMES ARE

h26id h26age h26sex h26ISI h26motor h26motorbi h26cog

h26cogbi h26job h26kei h26smk h26sake h26slep h26sleph

h26nut h26byoco h26byoco_bi h29ISI h29motor h29motorbi

h29cog h29cogbi h29chisy h29kei h29smk h29sake h29slep

h29sleph h29nut R2ISI R2motor R2motorbi R2cog R2cogbi

R2chisyu R2kei r2smk r2sake r2slep r2sleph r2nut

r2byoco r2byobi;

USEVARIABLES ARE

si14 si17 si20

pf14 pf17 pf20

scd14 scd17 scd20

age sex job kei smk sake byoco;

IDVARIABLE = h26id;

MISSING ARE ALL (-9999);

CATEGORICAL ARE

pf14 pf17 pf20

scd14 scd17 scd20;

DEFINE:

si14 = h26ISI;

si17 = h29ISI;

si20 = R2ISI;

pf14 = h26motorbi;

pf17 = h29motorbi;

pf20 = R2motorbi;

scd14 = h26cogbi;

scd17 = h29cogbi;

scd20 = R2cogbi;

age	= h26age;

sex	= h26sex;

job	= h26job;

kei	= h26kei;

smk	= h26smk;

sake = h26sake;

byoco = h26byoco;

STANDARDIZE age byoco;

ANALYSIS:

ESTIMATOR = BAYES;

CHAINS = 4;

PROCESSORS = 16;

FBITERATIONS = 320000;

THIN = 10;

BSEED = 2026;

MODEL:

! Parallel latent-basis growth processes: loadings 0 / free / 1.

i_si s_si |

si14@0

si17*

si20@1;

i_pf s_pf |

pf14@0

pf17*

pf20@1;

i_scd s_scd |

scd14@0

scd17*

scd20@1;

[i_si s_si i_pf s_pf i_scd s_scd];

i_si s_si i_pf s_pf i_scd s_scd*;

! Continuous ISI indicator intercepts fixed at zero.

[si14@0 si17@0 si20@0];

! Binary probit thresholds fixed at zero for identification.

[pf14$1@0 pf17$1@0 pf20$1@0];

[scd14$1@0 scd17$1@0 scd20$1@0];

! All pairwise growth-factor covariances.

i_si WITH s_si i_pf s_pf i_scd s_scd;

s_si WITH i_pf s_pf i_scd s_scd;

i_pf WITH s_pf i_scd s_scd;

s_pf WITH i_scd s_scd;

i_scd WITH s_scd;

! Seven baseline covariates predict all six growth factors.

i_si s_si i_pf s_pf i_scd s_scd ON

age sex job kei smk sake byoco;

! Estimate baseline covariate means and variances.

[age sex job kei smk sake byoco*];

age sex job kei smk sake byoco*;

! Label the PF slope-factor mean for the non-default prior.

[s_pf] (spf_int);

MODEL PRIORS:

spf_int ~ N(0.5, 1.0);

OUTPUT:

TECH1 TECH8 STDYX CINTERVAL;
