# Supplementary material for: Longitudinal co-development of social interaction, physical frailty, and subjective cognitive decline among community-dwelling older adults in Japan: a six-year parallel growth study
Source: Front Public Health. 2026 Sep 9;14:1913236. doi: 10.3389/fpubh.2026.1913236 (PMC13603030; doi:10.3389/fpubh.2026.1913236)
Supplement: Supplementary file 2 [file Supplementary_file_2.DOCX]

**Supplementary Table S1. Baseline characteristics of retained and non-retained participants**

| **Characteristic** | **Baseline cohort** | **Non-retained** | **Retained** | **SMD** |
| --- | --- | --- | --- | --- |
| Age, years | 74.28 ± 7.28 | 75.68 ± 7.66 | 71.64 ± 5.61 | −0.601 |
| Chronic disease count | 1.44 ± 1.17 | 1.48 ± 1.23 | 1.36 ± 1.04 | −0.103 |
| Index of Social Interaction score | 16.10 ± 2.51 | 15.72 ± 2.76 | 16.82 ± 1.75 | 0.473 |
| Female | 471/887 (53.1%) | 303/580 (52.2%) | 168/307 (54.7%) | 0.050 |
| Currently working | 320/764 (41.9%) | 178/491 (36.3%) | 142/273 (52.0%) | 0.317 |
| Good subjective economic status | 689/824 (83.6%) | 443/529 (83.7%) | 246/295 (83.4%) | −0.010 |
| Current smoking | 84/793 (10.6%) | 57/501 (11.4%) | 27/292 (9.2%) | −0.070 |
| Current drinking | 262/832 (31.5%) | 153/532 (28.8%) | 109/300 (36.3%) | 0.162 |
| Physical frailty (PF) | 163/884 (18.4%) | 136/577 (23.6%) | 27/307 (8.8%) | −0.401 |
| Screening-defined SCD | 340/887 (38.3%) | 244/580 (42.1%) | 96/307 (31.3%) | −0.224 |

*Note.* The baseline analytical cohort included 887 participants; 307 were retained in the three-wave sample and 580 were not retained. Continuous variables are mean ± SD; categorical variables are n/N (%) using available observations. SMDs are oriented as retained minus non-retained. SCD = screening-defined subjective cognitive decline based on the three-item KCL Cognitive Function domain.

**Supplementary Table S2. Baseline covariate effects and residual variances of growth factors**

**Panel A. Standardized baseline covariate effects on growth factors**

| **Growth factor** | **Baseline covariate** | **Standardized estimate** | **95% CrI** |
| --- | --- | --- | --- |
| i-SI | Age | −0.212 | −0.351 to −0.067 |
| i-SI | Sex | 0.009 | −0.145 to 0.163 |
| i-SI | Employment status | 0.159 | 0.010 to 0.304 |
| i-SI | Subjective economic status | 0.010 | −0.129 to 0.148 |
| i-SI | Current smoking | −0.135 | −0.275 to 0.009 |
| i-SI | Current drinking | 0.092 | −0.061 to 0.243 |
| i-SI | Chronic disease count | −0.008 | −0.147 to 0.131 |
| s-SI | Age | −0.369 | −0.587 to −0.132 |
| s-SI | Sex | −0.005 | −0.264 to 0.252 |
| s-SI | Employment status | 0.258 | 0.009 to 0.494 |
| s-SI | Subjective economic status | 0.056 | −0.177 to 0.286 |
| s-SI | Current smoking | −0.105 | −0.345 to 0.136 |
| s-SI | Current drinking | −0.132 | −0.383 to 0.123 |
| s-SI | Chronic disease count | −0.069 | −0.298 to 0.163 |
| i-PF | Age | 0.382 | 0.195 to 0.552 |
| i-PF | Sex | 0.091 | −0.128 to 0.308 |
| i-PF | Employment status | −0.099 | −0.320 to 0.129 |
| i-PF | Subjective economic status | −0.093 | −0.261 to 0.093 |
| i-PF | Current smoking | 0.058 | −0.152 to 0.258 |
| i-PF | Current drinking | −0.063 | −0.295 to 0.166 |
| i-PF | Chronic disease count | 0.243 | 0.046 to 0.426 |
| s-PF | Age | 0.493 | 0.234 to 0.707 |
| s-PF | Sex | 0.081 | −0.127 to 0.350 |
| s-PF | Employment status | −0.038 | −0.317 to 0.292 |
| s-PF | Subjective economic status | −0.106 | −0.324 to 0.142 |
| s-PF | Current smoking | −0.219 | −0.520 to 0.078 |
| s-PF | Current drinking | 0.073 | −0.197 to 0.418 |
| s-PF | Chronic disease count | 0.063 | −0.258 to 0.307 |
| i-SCD | Age | 0.103 | −0.129 to 0.331 |
| i-SCD | Sex | 0.145 | −0.111 to 0.396 |
| i-SCD | Employment status | 0.099 | −0.150 to 0.346 |
| i-SCD | Subjective economic status | −0.133 | −0.350 to 0.090 |
| i-SCD | Current smoking | −0.063 | −0.308 to 0.179 |
| i-SCD | Current drinking | −0.095 | −0.347 to 0.159 |
| i-SCD | Chronic disease count | 0.051 | −0.172 to 0.271 |
| s-SCD | Age | 0.308 | −0.007 to 0.594 |
| s-SCD | Sex | −0.127 | −0.469 to 0.208 |
| s-SCD | Employment status | 0.067 | −0.263 to 0.391 |
| s-SCD | Subjective economic status | −0.017 | −0.318 to 0.287 |
| s-SCD | Current smoking | 0.253 | −0.077 to 0.571 |
| s-SCD | Current drinking | −0.089 | −0.415 to 0.256 |
| s-SCD | Chronic disease count | 0.236 | −0.065 to 0.520 |

*Note.* Age and chronic disease count were standardized in the model. Other covariates were coded as described in the Methods. CrI = credible interval.

**Panel B. Residual variances of growth factors**

| **Growth factor** | **Posterior median** | **95% CrI** |
| --- | --- | --- |
| i-SI | 1.742 | 1.338 to 2.235 |
| s-SI | 0.566 | 0.287 to 1.052 |
| i-PF | 1.883 | 0.774 to 4.666 |
| s-PF | 2.402 | 0.541 to 13.012 |
| i-SCD | 0.670 | 0.330 to 1.442 |
| s-SCD | 0.471 | 0.156 to 1.300 |

*Note.* These are residual/conditional growth-factor variances after adjustment for the baseline covariates.
